# An Analysis of 2.3 Million Participations in the Continuing Medical Education Program of a General Medical Journal: Suitability, User Characteristics, and Evaluation by Readers

**DOI:** 10.2196/jmir.6052

**Published:** 2017-04-03

**Authors:** Hildegard Christ, Jeremy Franklin, Reinhard Griebenow, Christopher Baethge

**Affiliations:** ^1^ Institute of Medical Statistics, Informatics and Epidemiology University of Cologne Cologne Germany; ^2^ Municipal Hospital Cologne-Merheim University of Cologne Cologne Germany; ^3^ Deutsches Ärzteblatt Editorial Offices Cologne Germany

**Keywords:** education, medical, continuing interactive tutorial, journal article

## Abstract

**Background:**

Physicians frequently use continuing medical education (CME) in journals. However, little is known of the evaluation of journal CME by readers and also user and participation characteristics. Deutsches Ärzteblatt, the journal of the German Medical Association, is distributed to every physician in Germany and regularly offers its readers CME articles. Therefore, it provides a unique opportunity to analyze a journal CME program directed at an entire population of physicians.

**Objective:**

The aim is to show key sociodemographic characteristics of participants, frequency and temporal distributions of participations, and to analyze whether the articles are suitable for a general medical audience, how physicians rate the CME articles, how successful they were in answering simple multiple-choice questions, and to detect distinct clusters of participants.

**Methods:**

Using obligatory online evaluation forms and multiple-choice questions, we analyzed all participations of the entire 142 CME articles published between September 2004 and February 2014. We compared demographic characteristics of participants with official figures on those characteristics as provided by the German Medical Association.

**Results:**

A total of 128,398 physicians and therapists (male: 54.64%, 70,155/128,393; median age class 40 to 49 years) participated 2,339,802 times (mean 16,478, SD 6436 participations/article). Depending on the year, between 12.33% (44,064/357,252) and 16.15% (50,259/311,230) of all physicians in the country participated at least once. The CME program was disproportionally popular with physicians in private practice, and many participations took place in the early mornings and evenings (4544.53%, 1,041,931/2,339,802) as well as over the weekend (28.70%, 671,563/2,339,802). Participation by specialty (ranked in descending order) was internal medicine (18.25%, 23,434/128,392), general medicine (16.38%, 21,033/128,392), anesthesiology (10.00%, 12,840/128,392), and surgery (7.06%, 9059/128,392). Participants rated the CME articles as intelligible to a wider medical audience and filling clinically relevant knowledge gaps; 78.57% (1,838,358/2,339,781) of the sample gave the CME articles very good or good marks. Cluster analysis revealed three groups, one comprised of only women, with two-thirds working in private practice.

**Conclusions:**

The CME article series of Deutsches Ärzteblatt is used on a regular basis by a considerable proportion of all physicians in Germany; its multidisciplinary articles are suitable to a broad spectrum of medical specialties. The program seems to be particularly attractive for physicians in private practice and those who want to participate from their homes and on weekends. Although many physicians emphasize that the articles address gaps in knowledge, it remains to be investigated how this impacts professional performance and patient outcomes.

## Introduction

Proof of continuing medical education (CME) has become mandatory for physicians in many countries across the world. For example, in Germany proof of CME is required by law for those who finished residency training. As a result, medical specialists have to show that they continue learning.

The type of CME through which physicians in Germany satisfy this requirement is largely left open to the physicians themselves. Certified courses offered by various institutions (eg, hospitals, medical associations, private providers) are wide-ranging in form. They include lectures, symposia, sitting-in at a clinic, workshops, and structured interactive courses via print and online media, as defined by the Model Regulations on Continuous Medical Education and Certification of the German Medical Association. The Chambers of Physicians accredit a wide range of CME activities [1].

Although data on the “CME mix” (live events, print media, e-learning) as practiced by the individual physician are lacking, there are some data on the use of CME modalities in general on the national level. In 2015, all German Chambers together accredited approximately 360,000 CME activities. CME in print media constitute approximately 1% of all CME activities. However, this type of CME generated approximately 20% of all CME points earned by German physicians, demonstrating wide acceptance of this print article-based CME. For comparison, live CME events represented approximately 95% of all activities accredited, but generated approximately 70% of all CME points. This situation has shown only minor variations since these data have been recorded in 2006 (written personal communication, Reinhard Griebenow, Member of the Senate for medical education of the German Medical Association, August 2016). Accordingly, many journals in Germany offer CME articles. In 2014, there were 75 medical journals with an impact factor, and CME activities were offered in 60% of these.

International data are scarce, but in the United States where CME activity is an educational offer that is planned, implemented, and evaluated in accordance with the Accreditation Council for Continuing Medical Education (ACCME) Accreditation Criteria, journal CME represented 4% of all CME courses without commercial support. However, journal CME contributed 9% to all physician interactions related to CME in 2014 [2].

### Potentials and Challenges of Journal Continuing Medical Education

Offering CME in print media has several advantages. In contrast to almost all other forms of CME, print articles are often peer reviewed. Also, print CME is accessible independent of time and place, and a wide variety of courses with considerable content diversity can be offered. The challenges facing print CME include very diverse (and sometimes unknown) backgrounds of participants, which makes it more difficult to tailor the course to the participants’ abilities and interests. Also, feedback and the possibility to raise questions from participants are lacking or delayed. Similarly, discussion among participants is restricted, although online lists and communities can be a substitute for direct interaction. Finally, there is less control over evaluation of the participants’ success.

Accredited CME in print media consists of an article accompanied by 10 multiple-choice questions, of which at least seven have to be answered correctly (see Multimedia Appendix 1). For quality control, readers are asked to give feedback on several aspects of the articles (eg, presence of a current treatment strategy on the part of the participant, comprehensibility of the paper, completeness). In addition, participants are asked to provide basic demographic data and information regarding their specialty and work setting. It is mandatory for participants to submit these data to receive CME credits.

So far, studies analyzing print CME programs on the basis of such or similar data have mainly been related to specialist journals with CME units addressing a certain group of qualified specialist physicians. They are also based on a limited number of participants. For instance, in 2004 [3] and in 2005 [4] participations in CME offered in specialty journals were evaluated in two doctoral theses. Participants gave largely positive ratings, including the appropriateness of the content for their specialty.

In contrast to specialty journals, a comprehensive analysis of a CME print program addressing the general medical audience is lacking. *Deutsches Ärzteblatt* (Christopher Baethge is chief scientific editor), the journal of the German Medical Association, offers an ideal setting for such a study because of its interdisciplinary nature and its unique geographical coverage: all physicians in Germany receive a copy of *Deutsches Ärzteblatt*. For international readers, English translations of all CME, original, and review articles, as well as all editorials and letters to the editors, are provided in *Deutsches Ärzteblatt International*, the global edition of *Deutsches Ärzteblatt*.

In *Deutsches Ärzteblatt*, 142 CME articles have been published since the start of accredited CME in *Deutsches Ärzteblatt* in September 2004 until February 2014. There was a mean of 16,000 participants per unit. Because personal details have to be provided to receive CME points, it can be assumed that data have been conscientiously and accurately recorded. Furthermore, this provides a good opportunity to analyze the characteristics and evaluations of the physicians. A pilot study showed the feasibility of such an analysis, although the data basis was small (only 37 CME articles were evaluated) [5-7].

Accordingly, this investigation aimed to describe the personal, professional, and participation characteristics of all participants in the CME program of the *Deutsches Ärzteblatt* between 2004 and 2014. Specifically, we aimed to (1) describe the participants in terms of age, gender, and professional position; (2) describe the success rate and the evaluation of the scheme by the participants; (3) estimate the percentage of German medical professionals using this CME scheme; and (4) identify participant subgroups using cluster analysis and to assess the frequency and temporal distribution of participation.

## Methods

This is a retrospective analysis of primary data based on the records of 142 accredited CME articles in *Deutsches Ärzteblatt*. Because membership in a Chamber of Physicians is mandatory for all licensed physicians in Germany and the subscription fee for *Deutsches Ärzteblatt* is covered by the membership fee for the Chamber, every German physician receives *Deutsches Ärzteblatt* for free, resulting in a circulation of approximately 450,000 copies as of the first quarter of 2015.

Topics and authors of the CME articles are selected by the scientific editorial board of *Deutsches Ärzteblatt*. All articles have to pass peer review and the accreditation process of the Chamber of Physicians North-Rhine, which demands that not only the content of the article, but also the article pages, have to be free of any commercial influence and advertisements. Terms used in the German health care system are explained in Multimedia Appendix 2.

A CME unit consists of an article and a corresponding knowledge test with 10 multiple-choice questions, in which only one of five answers is correct (with a pass mark of 70%). The articles can be read in the printed edition of the journal or on the Internet, but test participation always takes place online. During the period of this study, each test was available online for six weeks (since then it has been extended to 12 weeks). In addition to the assessment of knowledge, all participants had to answer the following evaluation form:

1. Time required to finish the course: up to 30 minutes / 31 to 45 minutes / 46 to 60 minutes / more than 60 minutes.
2. Prevalence of the topic in everyday clinical practice: “The course topic arises in my professional activities frequently / regularly / seldom / never / not relevant for me.”
3. Presence of a treatment strategy for the clinical problem addressed in the article: “For the diagnosis and treatment of the disease concerned, I had already before my involvement with the course: a fixed treatment regimen / unsolved problems / no fixed strategy / not relevant for me.”
4. Completeness of information: “From the point of view of daily practice, important aspects of the course topic were not mentioned / treated too briefly / overemphasized / very well presented / not relevant for me.”
5. Comprehensibility of the article: “The article is comprehensible to specialists only / comprehensible to all physicians.”
6. Difficulty of the test questions asked: “The questions can be answered by studying the article only / only with additional literature.”
7. Overall satisfaction with the course (1=very good; 6=insufficient).

In addition, the date and time of participation was recorded. The data were stored in a database by an independent data host. For comparison with official figures, data on demographic characteristics and on medical specialties were taken from official statistics of the German Medical Association (Bundesärztestatistik).

Data analysis was performed using the statistical software SPSS version 22.0.0. Nominal and ordinal data are presented in tables using frequencies and percentages, contingency tables or bar charts. To identify groups of participants, a two-step cluster analysis (maximum: 4 clusters; distance measure: loglikelihood; cluster criterion: Schwarz-Bayes criterion) was performed. In view of the large sample size and because this is a descriptive investigation, *P* values are not presented.

## Results

### Participants

Of the 128,398 participants 98.36% (126,302/128,398) were active physicians. Per year, between 12.33% (44,064/357,252) in 2013 and 16.15% (50,259/311,230) in 2006 of all licensed physicians had participated [8].

**Figure 1:**
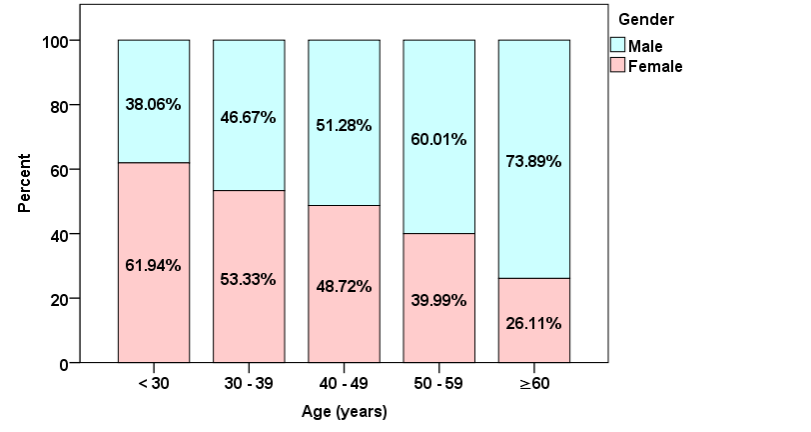
Age classes of German physicians participating in journal CME activities by gender.

The participants’ ages were distributed as follows: 5.78% (7420/128,393) were younger than 30 years; 21.34% (27,398/128,393) were aged between 30 and 39 years; 32.79% (42,096/128,393) were aged between 40 and 49 years; 28,51% (36,610/128,393) were between 50 and 59 years; and 11.58% (14,869/128,393) were 60 years or older. The age distribution showed a declining proportion of women with increasing age (see Figure 1).

**Figure 2:**
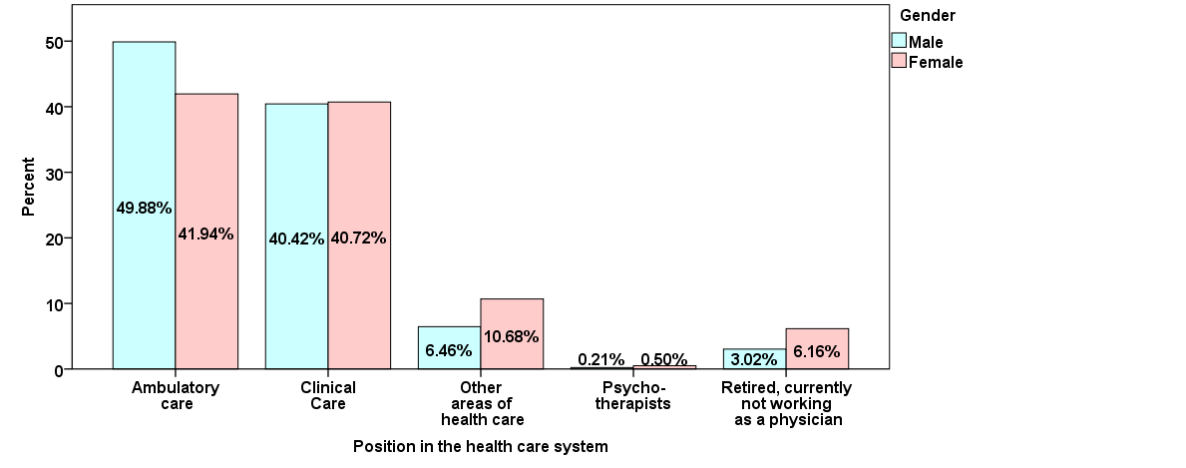
Positions in the health care system of German physicians participating in journal CME activities by gender.

The largest fraction of participants worked in private practice, with a larger proportion of men than women (8% more, see Figure 2). In all, 40.56% (52,069/128,390) of participants were employed in hospitals, with fewer women in senior positions (9.04%, 5265/58,237 vs 20.75%, 14,561/70,152). The proportion of residents who were female was higher (31.68%, 18,448/58,237 vs 19.66%, 13,795/70,152).

The most frequent specialty of all participating physicians was internal medicine with 18.25% (23,434/128,392), followed by general medicine (16.38%, 21,033/128,392), anesthesiology (10.00%, 12,840/128,392), and surgery (7.06%, 9059/128,392). In the cluster analysis, three groups of participants could be identified. The variables age, gender, position in the health care system, specialty, number of participations, median handling time (article and multiple-choice questions, hours), and mean number of CME points earned per course were employed. Age and gender emerged as the most strongly heterogeneous variables for characterizing the clusters (see Table 1).

**Table 1:** Cluster analysis of the participants.

Characteristics		Cluster 1	Cluster 2	Cluster 3
Participants, n (%)		55,101 (42.92)	37,873 (29.50)	35,415 (27.58)
**Gender, n (%)**				
	Male	55,101 (100)	0 (0)	15,051 (42.50)
	Female	0 (0)	37,873 (100)	20,364 (57.50)
**Age (years), n (%)**				
	<40	5249 (9.53)	5952 (15.72)	23,616 (66,68)
	40-60	39,235 (71.21)	28,354 (74.87)	11,115 (31.39)
**Position in health care system, n (%)**				
	Outpatient care	34,821 (63.19)	24,606 (64.97)	329 (0.93)
	Senior specialist	11,730 (21.29)	4942 (13.05)	57 (0.16)
	Other areas of health care	420 (8.02)	7278 (19.22)	1437 (4.06)
	Residents	1 (0.00)	0 (0)	33,531 (94.63)
**Specialty, n (%)**				
	Internal medicine	9858 (17.89)	4717 (12.45)	8859 (25.01)
	General medicine	9988 (18.13)	8214 (21.69)	2831 (7.99)
	Anesthesiology	4411 (8.01)	2937 (7.75)	5491 (15.50)
	Surgery	4490 (8.15)	983 (2.60)	3586 (10.13)
Participations, mean (SD)		20.00 (27.20)	21.26 (27.48)	12.21 (19.54 )

One cluster consisted of male physicians, mainly aged between 40 and 60 years, with 63.19% (34,821/55,101) working in outpatient care and 21.29% (11,730/55,101) being senior specialists.

A further cluster of exclusively female physicians had a similar composition, but with a lower fraction of senior specialists; instead, nearly one-fifth (19.22%, 7278/37,873) were occupied in other institutions.

The third cluster consisted almost exclusively of residents, 66.68% (23,616/35,415) being younger than 40 years and more than half (57.50%, 20,364/35,415) of them were women.

The mean number of course participations in cluster 1 was mean 21.26 (SD 27.48), in cluster 2 mean 21.26 ( 27.48), and in cluster 3 mean 12.21 (SD 19.54).

**Figure 3:**
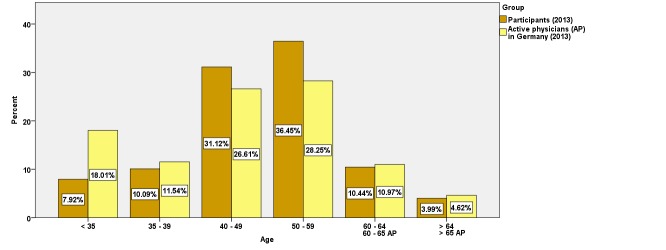
Age of CME participants compared with all active physicians in Germany.

Using the example from 2013, a comparison of the most important demographic data of the participants with the active physicians of Germany is given. Of the 357,252 active physicians in Germany, 44,064 (12.33%) were participants [8]. The proportion of women among CME participants (46.46%, 20,471/44,064) was very similar to that among all physicians in Germany (45.03%, 160,869/357,252). The proportion of those younger than 35 years among CME participants (7.92%, 3489/44,063) was less than half of that among all physicians in Germany (18.01%, 64,355/357,252; see Figure 3), whereas the participants aged between 40 and 60 years were overrepresented (67.56%, 29,772/44,063 vs 54.86%, 195,983/357,252).

**Figure 4:**
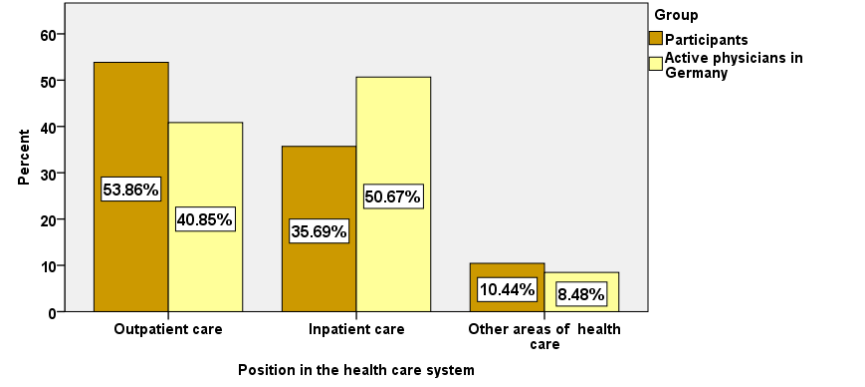
Position in health care system of CME participants compared with all active physicians in Germany.

The proportion employed in inpatient care was 14.68% lower among CME participants (35.69%, 15,728/44,064) compared with all physicians in Germany (50.67%, 181,012/357,252; see Figure 4).

**Table 2:** Articles with highest and lowest numbers of participants between September 2004 and February 2014.

Article		Participants, (n)	Overall satisfaction, mean (SD)	Questions correctly answered, mean (SD)
**Top ranking**				
	“Lyme borreliosis: current state of knowledge”	25,886	1.75 (0.68)	9.83 (0.46)
	“The acute abdomen from the internists’ point of view”	25,547	1.87 (0.68)	9.86 (0.46)
	“Key symptom diarrhea”	25,494	2.21 (0.83)	9.32 (0.83)
**Lowest ranking**				
	“Asperger syndrome: a disorder of the autism spectrum”	2407	2.17 (0.86)	8.98 (1.26)
	“Unwanted weight loss in the elderly”	2401	2.05 (0.82)	8.96 (0.85)
	“Obligatory reporting of infectious diseases”	2341	2.01 (0.85)	8.95 (1.06)

### Course Participations

The number of participants in each of the 142 CME courses published between September 2004 and February 2014 ranged from 2341 to 25,886 (mean 16,477, SD 6436). Table 2 displays the articles with the highest and lowest numbers of participants, respectively.

During this period there were 2,339,802 participations by 128,398 physicians and therapists (female: 45.68%, 1,068,559/2,339,032; male: 54.32%, 1,270,473/2,339,032) whose courses had been evaluated and whose data could therefore be analyzed.

The analysis of number of participations considered both inter- and intraindividual differences because 81.09% (104,122/128,398) of the participants took part more than once. For instance, both the workloads of the various participants in a course unit and the workloads of the same participant in various course units were analyzed.

Concerning participation frequency, approximately one-third (30.00%, 38,522/128,398) of participants each read 1 to 2, 3 to 10 (29.55%, 37947/128,398), and 11 to 50 articles (29.15%, 37,422/128,398), respectively; 11.30% (14,507/128,398) of participants read more than 50 articles. Of those participating 1 to 10 times, 29.47% (11,352/38514) were residents versus 19.03% (9883/51,929) of those participating more than 10 times.

Participations were recorded for all days of the week, although most frequently on Sundays and Mondays (see Table 3).

**Table 3:** Days of participations in journal CME activities.

Day	n (%)
Monday	394,232 (16.85)
Tuesday	345,400 (14.76)
Wednesday	334,516 (14.30)
Thursday	298,312 (12.75)
Friday	295,779 (12.64)
Saturday	244,447 (10.45)
Sunday	427,116 (18.25)

**Figure 5:**
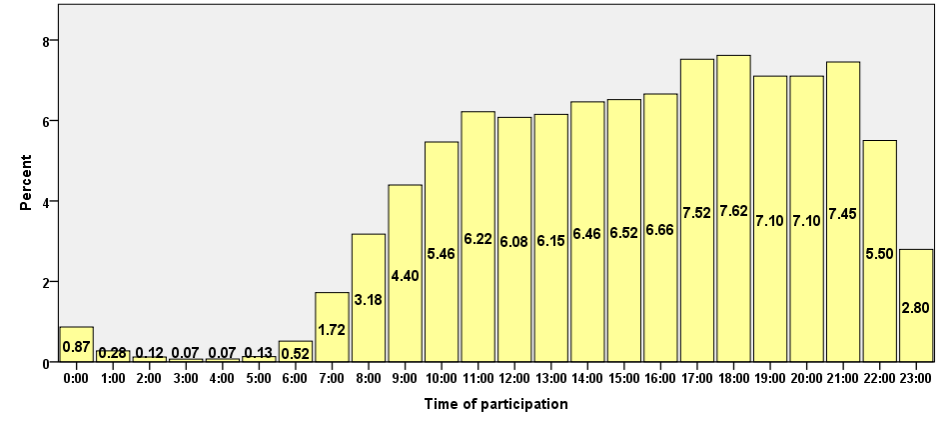
Time of day of participation in journal CME activities.

Although the great majority of participations (90.25%, 2,111,675/2,339,802) took place between 09:00 and 22:00 hours, 3.66% (85,702/2,339,802) occurred between 23:00 and 24:00 hours, 1.18% (27,809/2,339,802) between 01:00 and 06:00 hours, and 4.89% (114,616/2,339,802) between 07:00 and 08:00 hours (see Figure 5).

The temporal distribution of participations mirrors the general working times of the various sectors of health care. The proportion of physicians working in hospitals or being retired was larger at nighttime. Physicians not active in patient care tended to participate from 06:00 to 09:00.

More than three-quarters of participants (78.56%, 1,838,358/2,339,781) ranked the CME article as 1 or 2 (1=very good, 6=insufficient) and the knowledge test was passed by 99.07% (2,318,040/2,339,781) of participants (see Table 4). Between participations with ratings of 1 and 6, there was a difference of 6 percentage points in pass rates.

The materials were rated as “intelligible for all physicians, not only for specialists” in 95.65% (2,236,963/2,338,664) of participations. In 92.36% (2,156,478/2,334,986) of participations, the knowledge questions could be answered by studying the article materials only. However, for psychological psychotherapists (0.16%, 3677/2,338,664 of all participants), this number was lower (84.96%, 3124/3677).

**Table 4:** Overall satisfaction and pass rates of participants taking CME courses.

Overall satisfaction^a^	Frequency, n (%)	Passed, n (%)
1	527,951 (22.56)	525,622 (99.56)
2	1,310,407 (56.01)	1,301,059 (99.29)
3	377,073 (16.12)	370,874 (98.36)
4	87,803 (3.75)	85,563 (97.45)
5	27,633 (1.18)	26,575 (96.17)
6	8914 (0.38)	8347 (93.64)

^a^ 1=very good; 6=insufficient.

**Figure 6:**
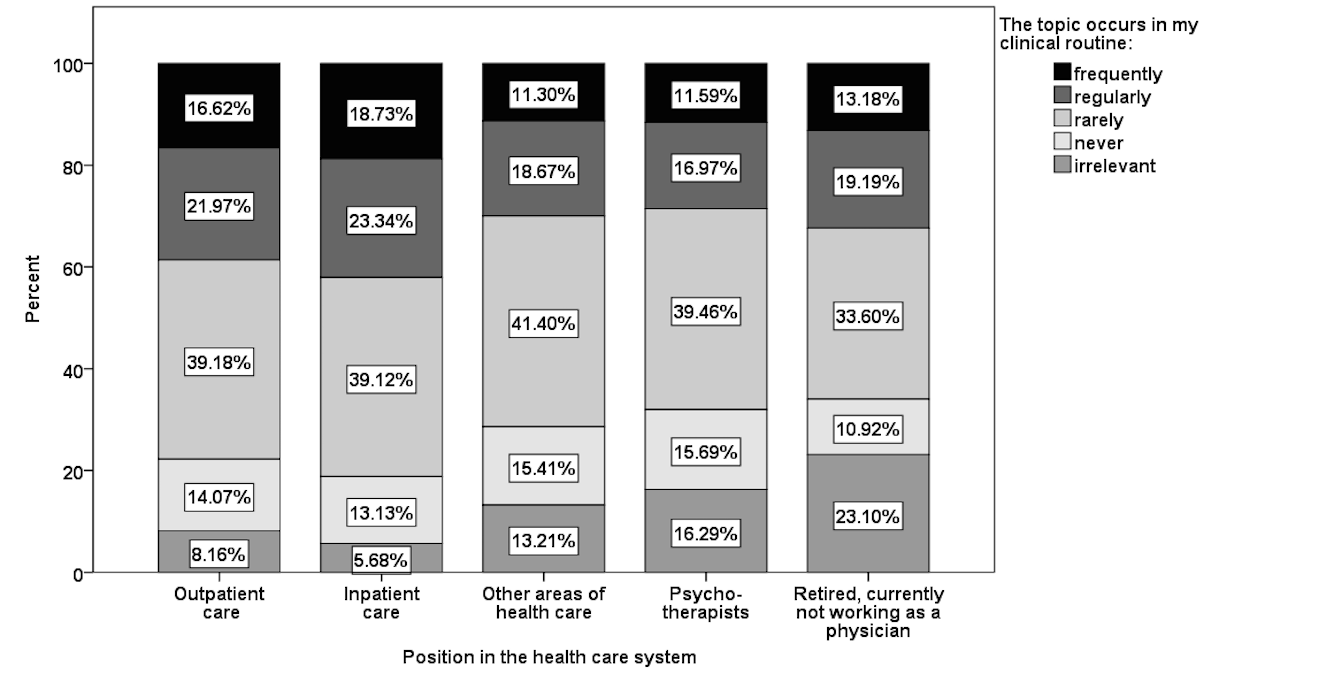
Representation of CME topics in clinical practice of participants.

The question “The topic occurs in my clinical routine” was answered “frequently” in 16.66% (389,624/2,33,9049) of participations, “regularly” in 21.99% (514,296/2,339,049), “rarely” in 39.17% (916,280/2,339,049), “irrelevant” in 13.71% (32,0724/2,339,049), and “never” in 8.74% (198,125/2,339,049) of participations (see Figure 6).

**Figure 7:**
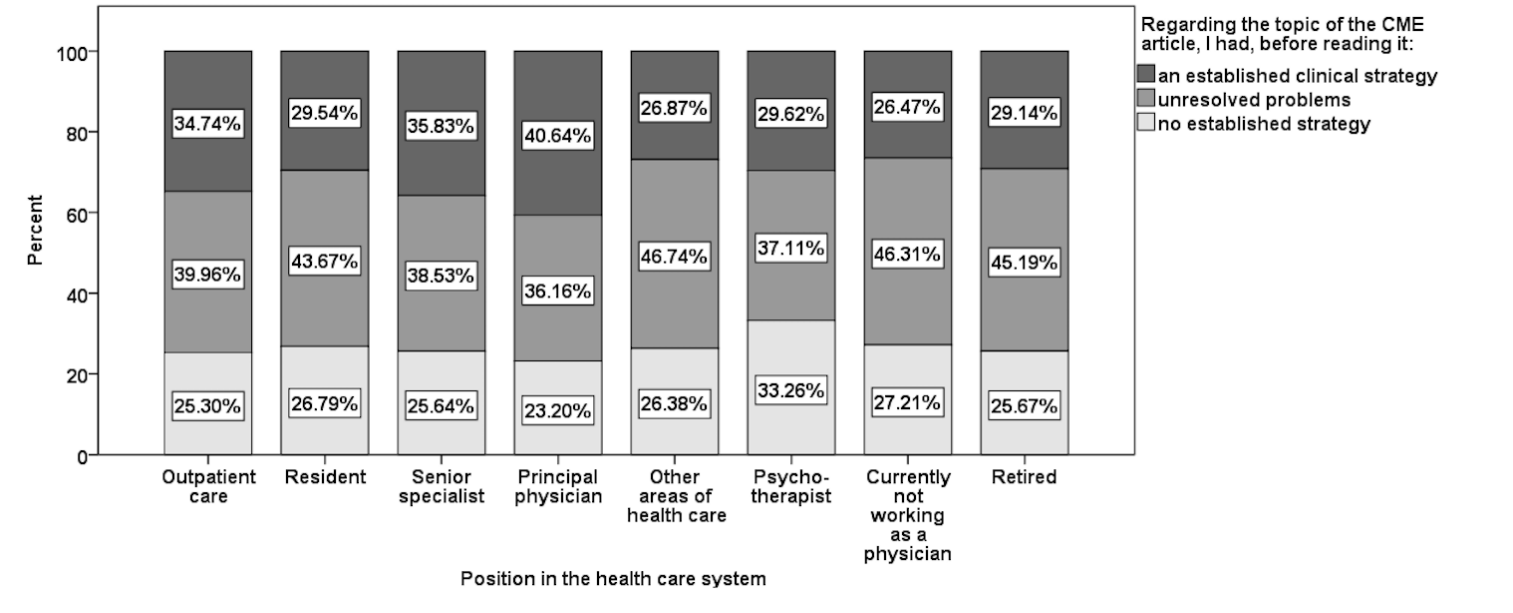
Presence of established clinical strategy, related to position in the health care system (“irrelevant” excluded).

In 25.61% (599,046/2,339,175) of participations, physicians had already “an established treatment strategy for the disease concerned” before reading the article, 32.88% (769,185/2,339,175) had “unresolved problems,” 20.44% (478,175/2,339,175) had “no established strategy,” and for 21.07% (492,769/2,339,175) the question was irrelevant. Physicians in private practice, senior specialists, and principal physicians showed the highest percentages for having a definite treatment regimen (see Figure 7).

**Figure 8:**
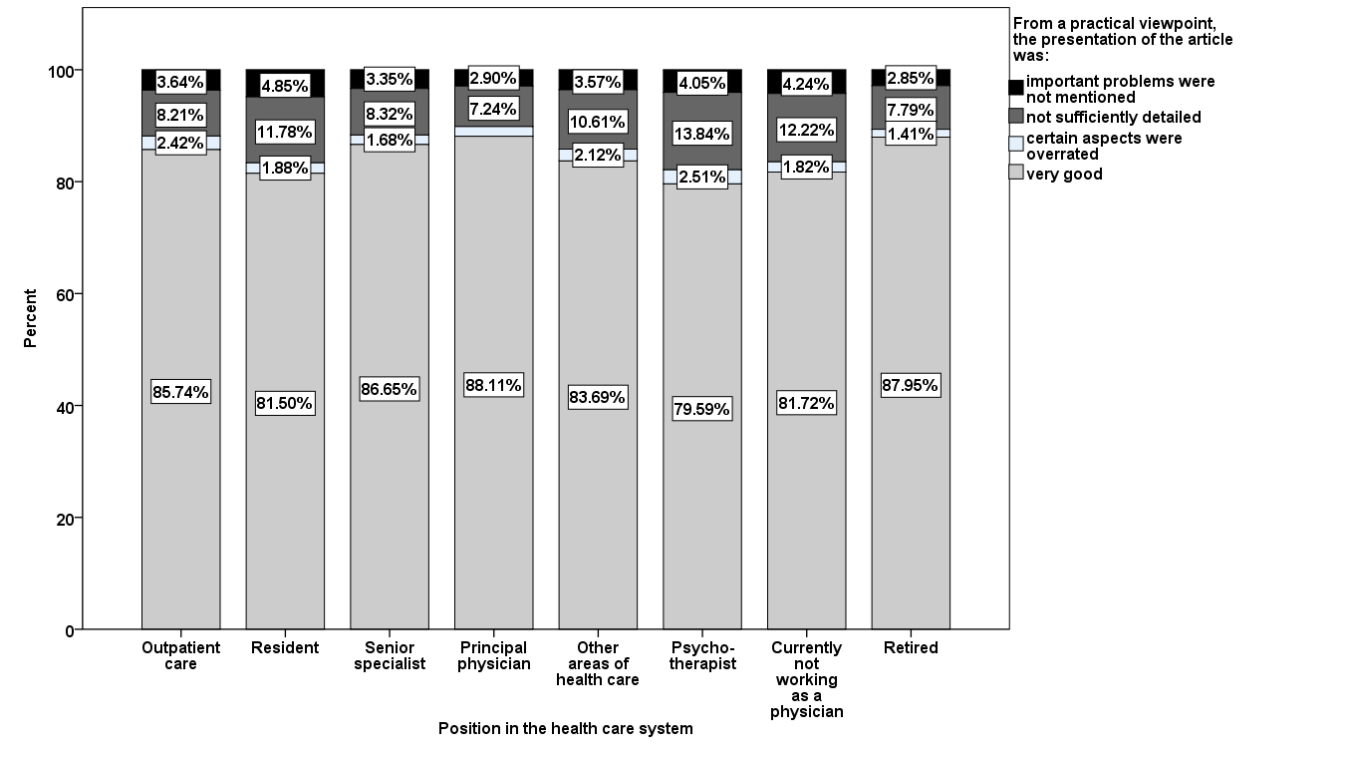
Balance of presentation of CME article content related to position in the health care system.

In 70.27% (1,643,825/2,339,275) of the participations, important issues were treated “very well” from the point of view of daily practice. Exclusion of participations that rated the content of the article as irrelevant for their medical practice increased this value to 84.44% (1,643,825/1,946,694). Physicians in private practice, principal physicians, senior specialists, and retired physicians gave higher ratings (see Figure 8).

## Discussion

This study yielded the following important results:

1. Interdisciplinary CME articles in *Deutsches Ärzteblatt* attract a significant percentage of all German physicians and were positively evaluated by the participants.
2. The time of day and day of week when participants worked on the articles as well as judgments on content indicate that CME in print media meets the needs of self-directed learning.
3. Three groups of participants in interdisciplinary CME articles in *Deutsches Ärzteblatt* could be identified, indicating distinct target audiences of print CME.

### Participants and Participations

The high participation rate in the CME articles of *Deutsches Ärzteblatt* supports the idea that print CME is an important pillar among CME programs. With regard to *Deutsches Ärzteblatt*, in any given year, approximately 15% of all licensed physicians take part. Moreover, most participants take part repeatedly, with one-third participating between 11 and 50 times over the years. Therefore, print CME contributes considerably to the sum of CME credits awarded to physicians obliged to prove continuing education. Of note, not only physicians obliged to collect CME credits take part: in our analysis, we found a cluster almost entirely consisting of residents, a group of physicians that under German law is exempt from collecting CME credits because residents are considered to be in a constant process of learning. Although the cluster of residents is the smallest cluster in our study, roughly one in four participants were grouped there, indicating that continuing education for physicians is not entirely driven by legal obligations.

Among a broad range of participants, we were able to identify two other well-defined groups, with age and gender being key characteristics. One cluster consisted of male physicians mainly in outpatient care, and a second cluster comprised of female physicians occupied in various fields (outpatient care, other areas of health care). It is obvious that the clusters are not representative of the entirety of German physicians; for example, older and younger physicians are underrepresented among our participants.

### Evaluations and Knowledge Tests

In general, the participants’ evaluations demonstrated a high degree of satisfaction with the articles; almost 80% rated them as good or very good. For 70% of all participations, important aspects of the topic were considered to have been very well presented. In a similar vein, in 96% of all participations readers found the content understandable even to physicians not belonging to the specialty mainly addressed in the article. It is reassuring that in the majority of participations the topic of the article occurred at least rarely, and often regularly and frequently, in participants’ everyday work and that many participants reported unresolved problems in dealing with the clinical topics. The high marks for satisfaction and understandability show that by a well-defined editorial process it is possible to deliver useful CME articles on a long-term basis.

The knowledge test was passed in 99% of participations, and in more than 92% the questions could be answered without use of any other additional material than article content. In Germany, knowledge tests are considered as formative and supporting the main messages of the article rather than reflecting the whole content of the CME article; they are not intended to be exams in the strict sense [9]. Nevertheless, knowledge tests add an element of quality assurance unique (in Germany) to CME in print media.

### Meeting the Learning Needs of Physicians

Our data further demonstrate that CME in print media meets the needs of self-directed learning not only with regard to content, but also to day and time of “attendance.” Articles were read on all days of the week, most frequently on Sunday. Further, almost 40% of participations occur before 09:00 or after 18:00, which clearly distinguishes them from live meetings. These data suggest that flexibility regarding location and hours is one of the main advantages of the CME program in print media. Therefore, if compatibility of family and work remains important or becomes even more important in medicine, print CME certainly has an important place among the variety of CME programs.

Among hospital physicians, articles are read less frequently by residents than by senior specialists and principal physicians, reflecting the fact that only specialists are obliged by law to document their CME activities. This is further supported by the fact that 46% of all participants worked in outpatient care, whereas hospital physicians were underrepresented compared to their nationwide representation [8].

### Factors Contributing to a Successful Print Continuing Medical Education Program

Our experience with CME at the *Deutsches Ärzteblatt* suggests that the following factors are important for a successful print CME program: topic diversity, comprehensibility, active intellectual involvement (hands on approach), regularity, easy-to-reach hotline and maintenance, and well-crafted multiple-choice questions that are fair and achievable. The commitment needed on the part of the journal is substantial: the time and effort involved in producing a CME article in *Deutsches Ärzteblatt* far exceeds that of a normal narrative review article. Producing CME articles encompasses, for example, the selection of authors and introducing authors into the specifics of CME articles (eg, formulating learning objectives, take-home messages, or multiple-choice questions). For educational material, both editorial and peer review need to be particularly thorough, and all multiple-choice questions have to be pretested. In total, we estimate that, compared to an average review article, CME articles take twice as much time and effort to publish. As a result, we devote approximately one-sixth of our staff resources to CME alone.

### Limitations

This investigation is limited to participants of the *Deutsches Ärzteblatt* CME scheme from 2004 to 2014. The characteristics of nonparticipants and the reasons for nonparticipation lie outside our scope. It would be interesting to see whether there are certain groups of physicians that cannot not be convinced to take part in print CME and what could be done to accommodate their needs. One obvious disadvantage of print CME is its lack of interaction between experts and participants. Therefore, in the future we are planning to offer an online question-and-answer format. Another problem of print CME regards the test format; although other forms of knowledge evaluation, such as interviews, are superior to multiple-choice questions in many ways, the sheer number of participants renders the multiple-choice question format inevitable. Two publications have summarized the relevant factors for good multiple-choice questions, demonstrated an improvement from 2006 to 2012 in *Deutsches Ärzteblatt* and showed the journal to be in a leading position among German journals offering CME [10,11]. Further, the consequences of print CME for everyday clinical practice remain unclear because no follow-up data concerning the application of gained knowledge or abilities are available. This aspect should be investigated in future studies.

### Conclusions

In summary, this investigation into the largest print CME program in German-speaking countries indicates that delivery of interdisciplinary CME articles is feasible and attracts a broad range of physicians with regard to medical specialty, age, gender, and position in the health care system. Nevertheless, we have identified certain clusters of physicians for whom self-directed learning by reading CME articles may be especially attractive.

## Appendix

Multimedia Appendix 1 Example of knowledge test, Deutsches Ärzteblatt International 2016; 113: 616–26.

Multimedia Appendix 2 Explanation of German health care terms in English.

## Abbreviations

CME: continuing medical education
