# Supplementary material for: An Analysis of 2.3 Million Participations in the Continuing Medical Education Program of a General Medical Journal: Suitability, User Characteristics, and Evaluation by Readers
Source: J Med Internet Res. 2017 Apr 3;19(4):e49. doi: 10.2196/jmir.6052 (PMC5394262; doi:10.2196/jmir.6052)
Supplement: Multimedia Appendix 1 [file jmir_v19i4e49_app1.pdf]

Please answer the following questions to participate in our certified Continuing Medical Education program. Only one answer is possible per question. Please select the most appropriate answer.

Question 1

How is neuropathic pain defined?

a) Neuropathic pain is pain that arises periodically while a neuroma forms.
b) Neuropathic pain is pain that arises as the direct consequence of a disease or lesion of the central and/or peripheral somatosensory nervous system.
c) Neuropathic pain is a periodically arising, unilateral pain of the musculoskeletal system.
d) Neuropathic pain is a paroxysmal, strictly unilateral, extremely severe type of headache that is felt mainly behind the eye and affects men and women in a ratio of 3:1.
e) Neuropathic pain is pain that arises during palpation of the abdomen when the examiner's hand is suddenly pulled away.

Question 2

A patient presents to you with a complex regional pain syndrome. Which of the following is a correct designation of this patient's pain syndrome?

a) Phantom pain
b) Purely central neuropathic pain
c) Mixed pain
d) Purely peripheral neuropathic pain
e) Purely nociceptive pain

Question 3

What is a realistic goal for the treatment of neuropathic pain?

a) Recovery and maintenance of the ability to work
b) 10% pain reduction at most
c) Long-term total freedom from symptoms without analgesic medication
d) Complete regeneration of damaged nerve cells through electrical nerve stimulation
e) Short-term pain relief through physiotherapy

Question 4

Which of the following are common side effects of gabapentin?

a) Headache, sedation
b) Dry mouth, hypotension
c) Extrapyramidal movement disorders
d) Exhaustion and anorexia
e) Daytime sleepiness and dizziness

Question 5

What, in particular, should the patient be told about before drug treatment is begun, in order to improve compliance?

a) The drug's approval status
b) The drug's performance-improving effect
c) The possible delay before the drug takes effect
d) The frequency of administration
e) The cost of treatment

Question 6

What percentage of patients fail to respond adequately to treatment when all of the pharmacotherapeutic options have been exhausted?

a) 0–20%
b) 20–40%
c) 40–60%
d) 60–80%
e) 80–100%

Question 7

Which of the following is the main clinical manifestation of central sensitization (i.e., hyperexcitability of the spinal cord)?

a) Chronic phantom pain
b) Decompression of nerve compression
c) Better sleep
d) Lumbar fusion
e) Intensified spontaneous pain

Question 8

What is the clinical result of spontaneous activity in C nociceptors?

a) Central sensitization
b) Reduction of the activating calcium influx
c) Mechanical allodynia and hyperalgesia
d) Inhibition of norepinephrine uptake
e) Hypesthesia

Question 9

Which of the following is a drug of first choice for the treatment of chronic neuropathic pain?

a) Non-opioid analgesics
b) Mirtazapine
c) Tetracyclic antidepressants
d) Beta-adrenoceptor antagonists
e) Tricyclic antidepressants

Question 10

When, as a rule, should a drug for the treament of neuropathic pain be entirely discontinued?

a) When pain is relieved by more than 30%
b) After three weeks of treatment
c) After a successful drug taper
d) When mild side effects arise
e) When pain is reduced to < 3 on the Numerical Rating Scale (NRS)
