# Supplementary material for: An Analysis of 2.3 Million Participations in the Continuing Medical Education Program of a General Medical Journal: Suitability, User Characteristics, and Evaluation by Readers
Source: J Med Internet Res. 2017 Apr 3;19(4):e49. doi: 10.2196/jmir.6052 (PMC5394262; doi:10.2196/jmir.6052)
Supplement: Multimedia Appendix 2 [file jmir_v19i4e49_app2.pdf]

Explanation of terms used in the German health care system

English/German	***Description***
General medicine/<br>Allgemeinmedizin	Basic health care at first point of contact and long-term care for acute and general medical problems
General practitioner/<br>Hausarzt	Physician functioning as first point of contact and long-term care for acute and general medical problems.
German Medical Assembly/<br>Deutscher Ärztetag	A parlament of doctors representing the entirety of German physicians that convenes annually and elects political representatives and decides on topics relevant to the profession.
The German Medical Association/<br>Bundesärztekammer	The German Medical Association is the central organisation in the system of medical self-administration in Germany.<br>As the joint association of the State Chambers of Physicians (Landesärztekammer), it represents the interests of German physicians in matters relating to professional policy, and plays an active role in opinion-forming processes with regard to health and social policy and in legislative.
Inpatient care/<br>Stationäre Versorgung	Health care in hospitals/clinics, involving admitted patients
Outpatient care<br>Ambulante Versorgung	Health care outside of hospitals/clinics, mainly in private practices, as well as non-admitted outpatients in hospitals/clinics
Physician in private practice/<br>Niedergelassener Arzt	Doctor working in his/her own, independent practice (possibly shared with other physicians)
Principal physician/<br>Chefarzt	Physician with leading supervisory function , usually in clinic or other health center
Private practice/<br>Privatpraxis	Independent practice owned and managed by one or more physicians
Resident/<br>Assistenzarzt	Qualified physician without supervisory function<br>(US: resident; UK: junior doctor or house officer)
Senior Specialist/<br>Oberarzt	Physician with supervisory function, usually in clinic or other health center
